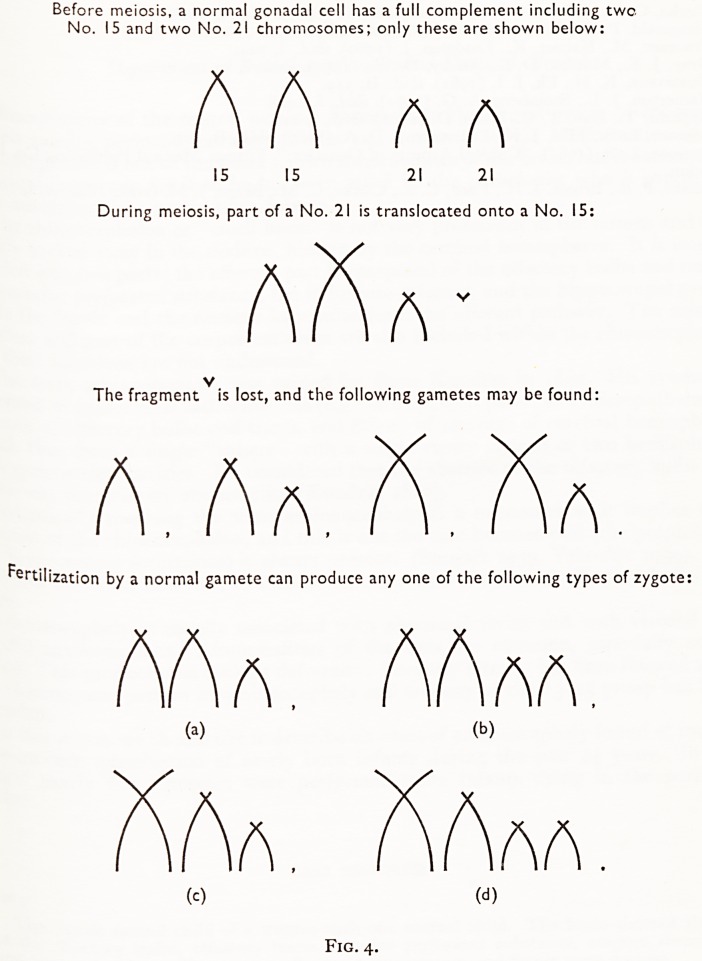# Translocation Mongolism Arising de Novo?

**Published:** 1965-01

**Authors:** P. W. Short


PLATE III

*Karyotype of translocation-mongol boy; note the abnormal large acrocentric chromosome. Below are the 13–15 groups from four other mitoses.*

### TRANSLOCATION MONGOLISM ARISING DE NOVO?

BY

P. W. SHORT

*Medical Student*

Mongolism has now been shown beyond doubt to arise as a result of the presence of extra genetic material from a small chromosome (No. 21) in the nuclei of cells of affected persons.

### TRISOMIC MONGOLS

The regular or trisomic mongol has 47 chromosomes, the extra one being a third No. 21. This chromosome appears in the cells as a result of non-disjunction, occurring usually at meiosis and occasionally during subsequent somatic divisions. Non-disjunction occurs at metaphase, when both daughter 21-chromosomes becomes included in the same germ cell. The other daughter cell thus has no No. 21-chromosome, and is presumably non-viable, as no metaphase plate has yet been seen with this abnormality (Fig. 1).

Formation of Gametes

(a) Non-disjunction at first meiotic division: formation of two gametes with an extra chromosome.

(b) Non-disjunction at second meiotic division: formation of two normal gametes, and one gamete with an extra chromosome.

FIG. 1.

If non-disjunction occurs during the first mitotic division of the zygote, one daughter cell will have 47 chromosomes, and the other one will have 45. The latter cell is not viable and the embryo will thus develop from the cell containing 47 chromosomes and be indistinguishable from a mongol produced as a result of an error during meiotic division (Fig. 2a). However, if non-disjunction occurs at a later mitotic division of a previously normal zygote, in which cell lines with 46 chromosomes are already established, new daughter cells with 45 and 47 chromosomes will again result; but as the cell with 45 chromosomes is non-viable, only one new line of cells will be established, and the resultant embryo will be a mosaic composed of its original set of cells with

46 chromosomes, together with a new set (arising from the abnormal division) with 47 chromosomes (Clarke *et al.*, 1961; Heyashi *et al.*, 1962) (Fig. 2b). It will be a matter of chance which body cells have the abnormal chromosomal constitution.

If non-disjunction occurs during a mitotic division of one of the cells of an already mongol embryo, the daughter cells resulting from this division will have 46 and 48 chromosomes respectively and the resultant embryo will be a more complicated mosaic with three cell lines, one with 46, one with 47, and one with 48 chromosomes (Fitzgerald *et al.*, 1961; Gustavson *et al.*, 1961) (Fig. 2c).

(a) Non-disjunction at the first somatic division of the zygote, resulting in the formation of an individual all of whose cells contain 47 chromosomes.

(b) Non-disjunction at a division subsequent to the first somatic division, resulting in a two-cell-line mosaic.

(c) Non-disjunction in a mongol embryo, at a division subsequent to the first somatic division, resulting in a three-cell-line mosaic individual.

FIG. 2.

### TRANSLOCATION MONGOLS

Polani *et al.* (1960), investigating three mongols selected for the youth of their parents, found that one of these infants had only 46 chromosomes. These cells on examination appeared to lack a chromosome in the 13–15 group and to have an extra chromosome in the 6–12 group. On examining the chromosomes of the mother, it was found that this pattern was repeated, but that in addition a No. 21-chromosome was lacking, and there were thus only 45 chromosomes. The father's chromosomal constitution appeared normal. From this it was inferred that the abnormal "6–12" chromosome was produced as the result of the translocation of a No. 21-chromosome to a member of the 13–15 group (generally accepted as being a No. 15). Thus the mother had the normal amount of genetic material, though it was distributed in 45 instead of 46 chromosomes. She was phenotypically normal, but could transmit to her offspring the entire translocated chromosome. Many similar cases have been reported (Hamerton *et al.*, 1962), and there have been others in which the translocation has occurred between the small acrocentric chromosomes (Nos. 21 and 22). But the regular, or trisomic, is by far the most common form of mongolism.

### TRANSLOCATION MONGOLISM APPEARING SPORADICALLY

At the Bristol Royal Infirmary we have recently been investigating the genetic constitution of mongol infants and their parents by the karyotyping of metaphase plates made from phytohaemagglutinin cultures of peripheral blood lymphocytes.

A translocation-mongol boy has been discovered (Plate III) whose parents are of normal chromosome constitution. Other similar cases have been reported (Fitzgerald *et al.*, 1961; Penrose *et al.*, 1960; Carter *et al.*, 1960). Penrose advanced the theory that one of the parents had a gonadal mosaicism for the 15/21 translocation, which would not of course be detected by the usual cultural techniques. A virtually similar theory was put forward by Polani (1960).

How can such a state of mosaicism be imagined to arise? We have to account for an individual with at least two cell-lines, of which one will be of the mongol translocation-carrier type, and one, the most numerous, will be normal.

(i) The zygote may initially be of translocation-carrier composition. For a normal cell-line to arise there would have to be three simultaneous non-disjunctions (shown as heavy lines in Fig. 3). This is an unlikely event, and even if it occurred the normal, and not a translocated line, would be in a minority in the individual.

(ii) It would seem easier to suppose that in a normal person a 15/21 translocation should occur at some time in a cell destined to form gonadal material; this is the explanation put forward by Polani (1960) and Penrose (1961). A woman in whom this occurred would presumably be phenotypically normal, and if the translocation was confined to ovarian tissue there would be little or no chance of detecting it. The main objections to this theory are:

(a) Translocations are generally considered to take place during the diplotene phase of meiosis, whereas if only the gonad is to be affected, in part or in whole, the translocation must occur during a mitotic division.

(b) No translocation mosaicism has yet been found.

(c) If the gonads are affected, it would be expected that other offspring in the family would carry the translocated chromosome. This has not yet been reported.

FIG. 3.

Thus the explanations that have been advanced, though possible, do not correspond with very probable events. A simpler explanation would be more acceptable. A clue to this may be given by considering how a translocation-carrier first originates. Carter *et al.* (1960) show a diagram explaining this (Fig. 4). We have seen that the translocation is thought to take place at meiotic diplotene, after which error there is a possibility of producing four types of gamete (Fig. 4). Theoretically, any one of these may fuse with a normal gamete at conception, so that any one of the four genetic types of individual (Fig. 4, a, b, c, d) may be produced. Type (a) is monosomic for chromosome 21, and would not survive. If type (b), a normal individual, was produced, no one would ever know that there had been an error at meiosis. If type (c) was produced, then a translocation-carrier line has been started, fulfilling the original object of the explanation. But there is a final possibility, the production of type (d), which is a translocation-mongol produced by genetically and phenotypically normal parents.

A line of translocation-carriers must start some time from a normal person, in the manner outlined above. Whenever the conditions arise that could start a carrier line, they could instead cause the appearance of a new translocation-mongol. Therefore

FIG. 4.

if we accept Carter's explanation for the pathogenesis of translocation-carriers we must also admit the possibility that translocation mongols may appear *de novo* as the offspring of normal parents. This explanation, which involves no new mechanism, is advanced to account for the present case.

### REFERENCES

Carter, C. O., Hamerton, J. L., Polani, P. E., Gunlap, A., Weller, S. D. V. (1960). *Lancet* **ii,** 678.

Clarke, C. M., Edwards, J. H., Smallpiece, V. (1961). *ibid.,* **i,** 1028.

Fitzgerald, P. H., Lycette, R. R. (1961). *ibid.,* **ii,** 212.

Fraccaro, M., Kaijser, K., Lindsten, J. (1960). *ibid.,* **i,** 724.

Gray, J. E., Mutton, D. E., Ashby, D. W., (1962). *ibid.,* **i,** 21.

Gustavson, K. H., Ek, J. I. (1961). *ibid.,* **ii,** 319.

Hamerton, J. L., Steinberg, A. G. (1962). *ibid.,* **i,** 1408.

Hayashi, T., Hsu, T. C., Chao, D. (1962). *ibid.,* **i,** 218.

Penrose, L. S., Ellis, J. R., Delhanty, J. D. A. (1960). *ibid.,* **ii,** 409.

Penrose, L. S. (1961). "Clinical Aspects of Genetics." Pitman Medical Publishing Co. Ltd. London.

Polani, P. E., Briggs, J. H., Ford, C. E., Clarke, C. M., Berge, J. M. (1960). *Lancet,* **i,** 721.

## Figures and Tables

**PLATE III f1:**
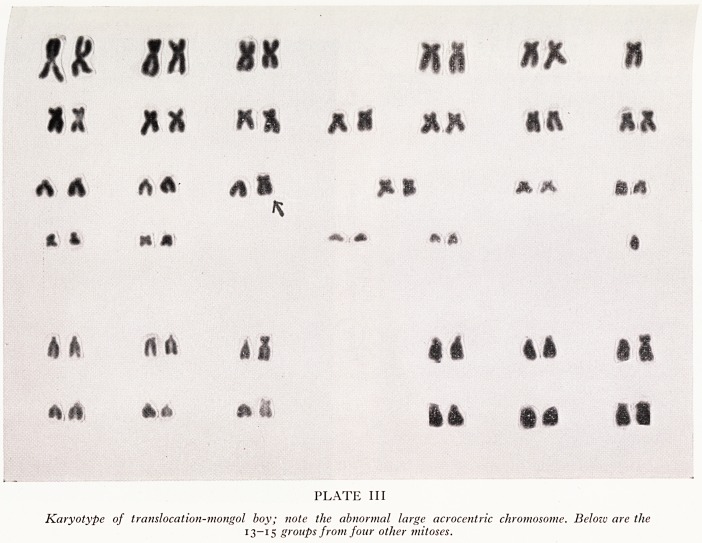


**Fig. 1. f2:**
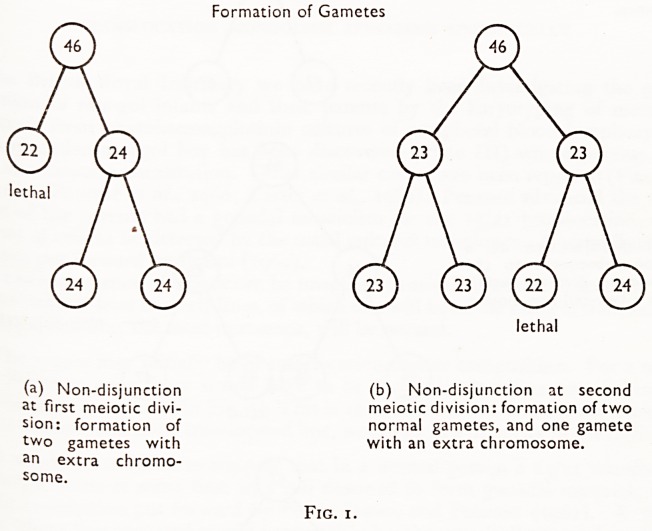


**Fig. 2. f3:**
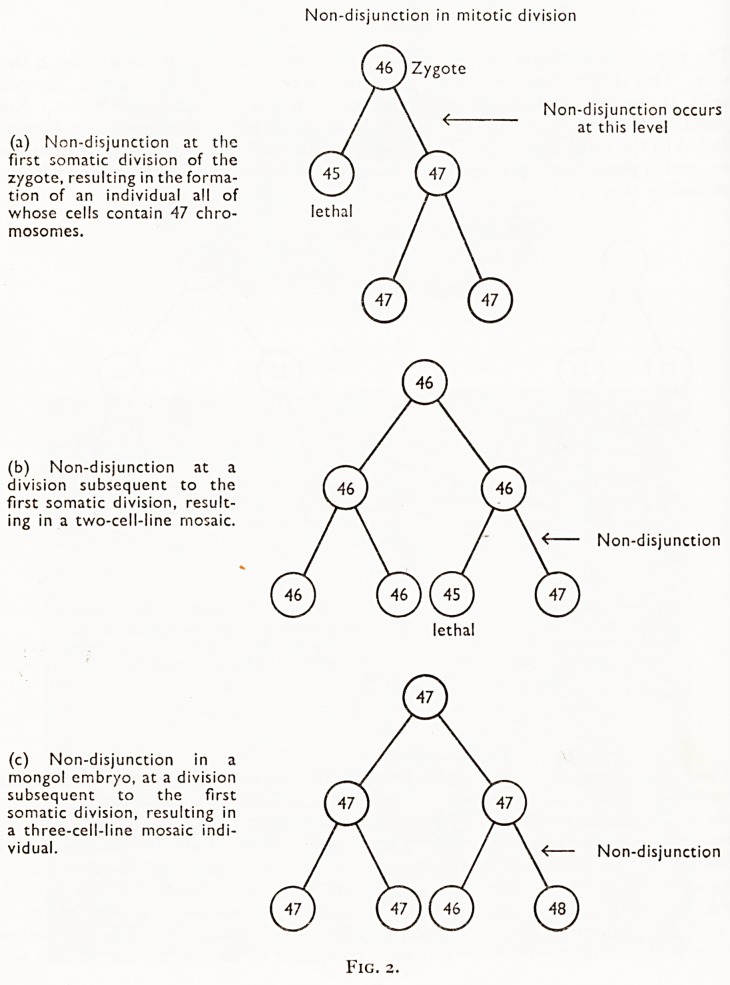


**Fig. 3. f4:**
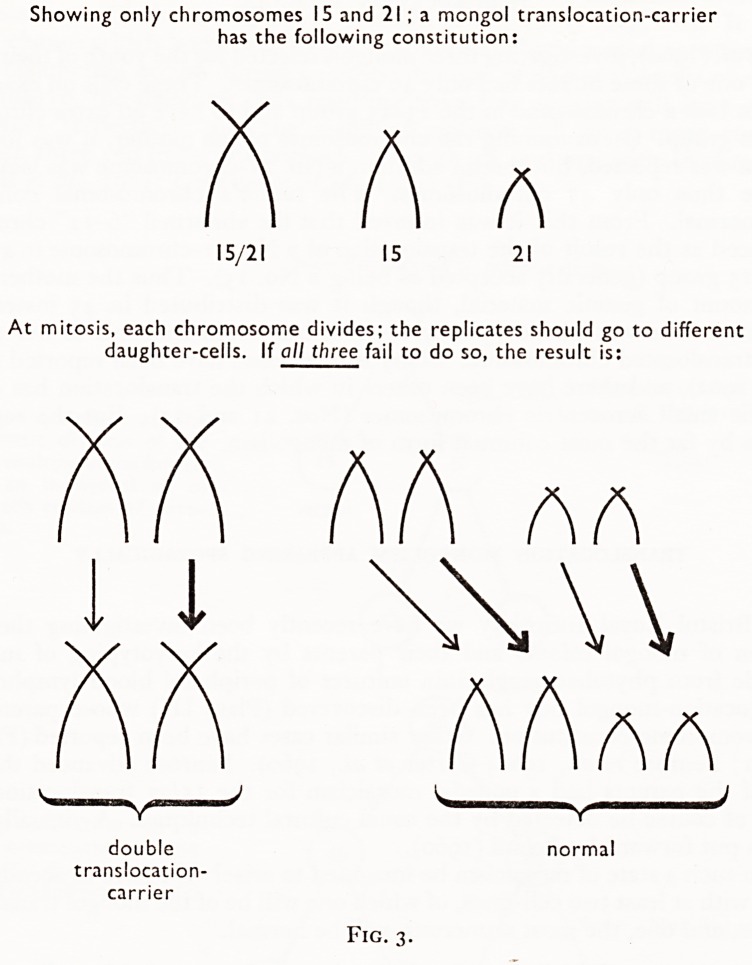


**Fig. 4. f5:**